# Digital Lengthening to Treat Finger Deficiency: An Experience of 201 Digits in 104 Patients

**DOI:** 10.1155/2017/4934280

**Published:** 2017-02-12

**Authors:** Zhenyu Ding, Xiaozhong Zhu, Kai Fu, Xianyou Zheng

**Affiliations:** Department of Orthopedics, Shanghai Jiaotong University Affiliated Sixth People's Hospital, 600 Yishan Road, Shanghai 200233, China

## Abstract

*Objectives.* We evaluated the results of digital lengthening by distraction and second-stage bone graft.* Methods.* We treated finger deficiency of 201 digits in 104 patients (68 males, 36 females) by digital distraction and second-stage bone graft. The distraction was performed with a rate of 1 mm/day (for the first ten days) and 0.5 mm/day followed by using a self-designed bilateral tubal-helical external fixator. The mean follow-up period was 42 months (range 6 to 60 months).* Results.* The mean lengthening was 29.2 mm (range 25 to 40 mm) and 18.7 mm (range 12 to 32 mm) for metacarpal bones and phalanges, respectively. The mean elongation rate was 174.4% (range 145% to 202%) and 184.8% (range 115% to 283%) for metacarpal bones and phalanges, respectively. The static two-point discriminations and SpO_2_ showed no significant differences before and after distraction. Four complications were observed (two skin ruptures and two phalangeal splitting). No pin tract infection or tendon rupture showed. Digital lengthening improved functions of the hand.* Conclusion.* Digital distraction and second-stage bone graft is an effective method to compensate disabilities caused by lack of finger length. It could be an alternative plan for patients with thumb deficiency instead of toe-to-thumb transplant and patients with finger deficiency instead of ray resection.

## 1. Introduction

Posttraumatic digital deficiency may result in partial or even complete loss of digital opposition function and the contour of the affected hand. One of the effective reconstructive surgeries is digital lengthening to improve opposition function.

In the past few decades, several reconstructive solutions were proposed to treat digital deficiency, including the tubular skin flap transfer, the toe-to-thumb transplant, and digital distraction with/without bone graft. Though the traditional tubular skin flap transferring and bone graft may ideally increase the length of the finger, the contour and function are not satisfying. Microsurgical toe-to-thumb transfer for the amputated thumb reconstruction will sacrifice a healthy toe and is difficult to operate despite a better appearance and function [1]. The metacarpal lengthening carried out in a single stage overcomes the above drawbacks, while the limited extended length may not cater the demands of the patients [2]. In orthopedic surgery, successful experiences of gradual distraction for joint contracture and osteoepiphysis distraction for shortening deformity of extremities demonstrate that not only soft tissues like vessels, nerves, and tendons but also the bone have potentials to extend correspondingly under tensile force [3]. Furthermore, the extension will not harm soft tissue itself if within permissive flexible range [4]. Accordingly, gradual digital distraction in the first stage and bone graft in the second-stage may be a better choice to treat digital deficiency.

## 2. Patients and Methods

**Figure 1:**
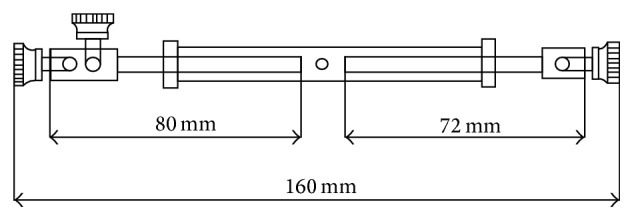
The digital external fixator was a tubal-helical distraction device. When rotating the cannula with the spanner, the bolts extended in opposite directions, of which maximum extended length reached 80 mm.

Since 1996, 201 residual digits of 104 patients underwent gradual distraction and bone graft with a self-designed digital external fixator (produced by Wujin No. 3 Medical Instruments Factory, Jiangsu Province of China) in our department (Figure 1). The 68 male and 36 female patients were aged 6 to 37 (average 29.5). The delay between initial injury and distraction operation ranged from 3 months to 12 years (average 30 months). According to the number of finger defects, 44, 31, 21, and 8 cases were one-digital, two-digital, three-digital, and four-digital deficiency individually. And concerning the location of the deficient digits, 31 thumbs, 59 index fingers, 65 middle fingers, 34 ring fingers, and 12 little fingers were involved. Based on the level of the deficiency, 6 digits, 167 digits, and 28 digits were located in the metacarpophalangeal joints, the proximal phalanges, and the middle phalanges individually. Eighteen thumbs and two index fingers underwent metacarpal lengthening while the remaining 181 fingers underwent phalangeal lengthening. The basic data of all patients was shown in Table 1.

**Figure 2:**
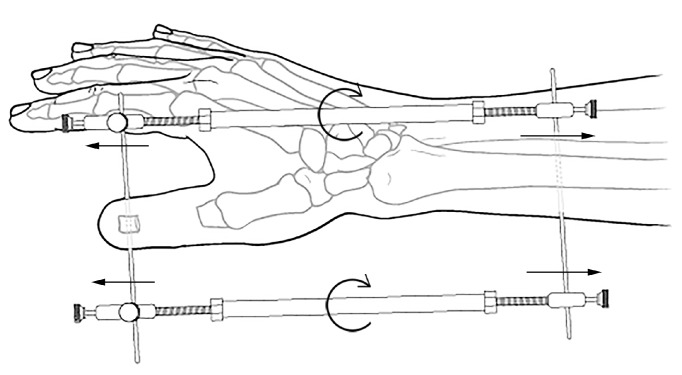
Lengthening of proximal phalange of thumb.

The operation was performed in two stages under brachial plexus block anesthesia. The first stage was digital lengthening. In the cases of single thumb deficiency, the first metacarpal osteotomy was undergone. We used a small osteotome to make a subperiosteal osteotomy between extensor pollicis longus and brevis tendons through a 2 cm longitudinal dorsal approach. In the cases of single finger deficiency without thumb involved, the phalangeal osteotomy was performed with a longitudinal incision in the dorsolateral of the affected phalange. After pulling the lumbrical tendon to the dorsal, the periosteum was cut-off to expose the phalange. A transversal K-wire was penetrated distal to the osteotomy level; then the metacarpal or the phalange was dissected. The digital external fixator was tuned to the minimum length to locate the penetrating spot of the radius on the skin. Another K-wire was penetrated through the radius percutaneously, parallel to the first wire. Then, we combined the K-wires with the external fixators (Figure 2). As for multiple fingers deficiency, two or more fixators might be added simultaneously. Intraoperatively, 4-5 mm width could be extended. Gradual distraction at the rate of 1 mm/day in the first 10 days and 0.5 mm/day in the following days was carried out postoperatively. The distraction process was terminated after achieving the desirable length in comparison to the contralateral normal side, usually after 17–40 days (average 26 days). The pin track was disinfected 2-3 times per day after operation. Patients at discharge were taught to perform the elongation himself carefully.

**Figure 3:**
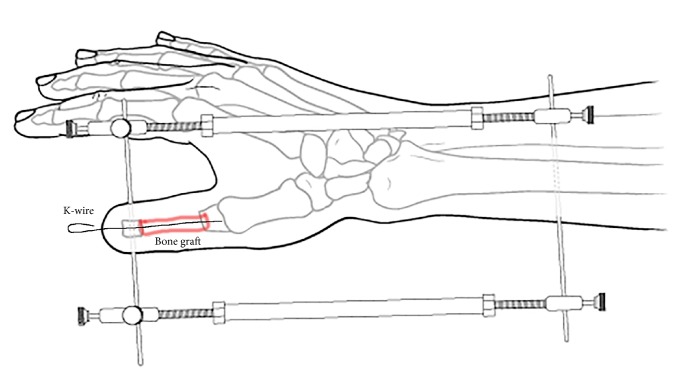
Bone graft was fixed with a K-wire.

After 2-3 weeks' distraction to achieve the suitable length, the second-stage bone graft was performed. Iliac crest autografts were used in 173 digits while the xenogeneic bone grafts were used in 28 digits. Through the original incision, the bone graft was inserted between the two osteotomy ends. After making a bolt-shaped end, the bone graft was embedded in the proximal medullary cavity and the other end was immobilized with a K-wire (Figure 3). The K-wire was pulled out when the bone healed. Additionally, first web deepening or finger web augmentation was performed in 36 cases with the Z shaped flaps or the dorsolateral flaps of the index finger after bone graft.

In the latest follow-up, the maximum elongation of the residual digits was measured with a straight edge, and the maximum elongation rate was calculated accordingly. The bone healing process was observed by X-ray films. The static two-point discrimination of the affected fingertip was measured with a compass before and after distraction. The saturation of blood oxygen (SpO_2_) of before and after lengthening was measured with finger pulse oxygen detector.

## 3. Statistical Analysis

Means and standard deviations were calculated from individual values using standard procedures by SPSS, version 22.0 (SPSS Inc., Chicago, IL, USA) statistical package for personal computers. Pairwise *T* test was used to determine significant differences between the pre- and postlengthening. *P* < 0.05 was regarded as statistically significant.

## 4. Results

The follow-up period ranged from 6 to 60 months (average 42 months). During distraction, complications of 4 digits were observed: two skin ruptures of the finger tips (one for a too fast distraction and the other for fragile skin of the stump) and two phalangeal splitting due to the K-wires being too close to the ends. And no infection or rupture of tendons occurred. Though ten patients showed swelling and redness around the pin tracks, close pin track care with/without antibiotics therapy prevented this from turning into infection. Postoperatively, the digital tips got good sensory and blood supply. X-ray films indicated that the bone healed with the graft.

The achieved elongation of the 20 metacarpal bones varied from 25 mm to 40 mm (average 29.2 mm), and of the 181 digital phalanges from 12 mm to 32 mm (average 18.7 mm). The elongation rates were 145%–202% (average 174.4%) of 20 metacarpal bones and 115%–283% (average 184.8%) of the 181 digital phalanges, respectively (Table 2).

Static two-point discriminations of the affected finger tips were 6.94 ± 0.77 mm before lengthening and 7.06 ± 0.84 mm after lengthening. The SpO_2_ of pre- and postlengthening was 96.47 ± 1.23% and 96.29 ± 1.34% individually. There were no significant differences of the static two-point discriminations and SpO_2_ between pre- and postlengthening (*P* > 0.05) (Table 3).

## 5. Discussion

The procedure is indicated for single or multiple digital deficiencies at the level of the proximal or middle phalange while the soft tissue is well covered. The site of the osteotomy is based on the position of the residual finger and the level of the deficiency. The first metacarpal bone osteotomy is carried out when the level of the residual thumb is located in the metacarpophalangeal joint or the base plane of the proximal phalange. The proximal phalange osteotomy is performed if the proximal phalange stump of the thumb is more than 15 mm in length. And the osteotomy of the other 4 fingers may be operated while the deficient level is located in the proximal phalange or middle phalange with a bone stump longer than 10 mm.

The lengtheners used presently such as Matev, mini-Hoffman, and Ikuta are mainly indicated for the metacarpal bone prolongation [5, 6]. However, the lengthener designed and applied in the present study was a tubal-helical distraction device, which might be used not only for the metacarpal bone prolongation but also for the phalangeal bone prolongation. Furthermore, different from the past ones, multiple devices might be combined for multidigit lengthening at the same or different deficient level.

The fixator in our study has great prolongation potentials as the long distance between the two K-wires and plenty of soft tissue. The maximum elongation rate in this trail was 283% consistent with Ivan Matev, who reported that after metacarpal lengthening, 92 thumb deficiency patients obtained length improvement that varied from 20 to 45 mm (average 35 mm) or equal to the length of the thumb proximal phalange [7].

Compared with the possible compression injury of the adjacent joints during the metacarpal lengthening, adjacent joints were under dragging force during the phalangeal lengthening in our trial. Thereby the risk of contracture of the collateral ligaments and compression injury of the articular surface was minimized and the affected digits gained better joint motion. In addition, the prolongation level was located in the distal of the footprint of the flexor tendon, so less dragging force was given directly to the flexor muscle than that during the metacarpal lengthening, which might cause ischemic contracture due to the excessive traction of the hand intrinsic muscles.

Consistent with other authors, we used second-stage bone graft in order to achieve early bone consolidation [8, 9]. In our trial, the osteotomy was performed under the periosteum; thus the integrality was retained. During the prolongation, the periosteum was gradually lengthened to strengthen osteogenesis, which might maintain the long-term length of the prolongated digit and minimize the bone resorption risk. Postoperative X-ray films indicated the consolidation of the grafted bone. Because no distinct difference could be found between the iliac crest and the xenogeneic bone graft, the preference of the latter might be implied in order to lessen the suffering caused by autogenous harvest. It has also been noted that some authors prefer spontaneous bone consolidation without bone graft. Seitz and Froimson [10] reported that 14 cases of lengthening for digital deficiency were carried out in single stage and provided between 20 mm and 35 mm lengthening per digit with a slow rate of lengthening (0.25 mm in four daily increments). However, only one case needed additional bone graft. Kömürcü, Bosch, and other authors also had satisfactory results without bone graft [11, 12]. Therefore, the use of bone graft is still controversial.

Compared with other digital lengthening methods, the disadvantages of this procedure include a two-stage operation, bone graft resorption risk, and long-time treatment. And the wrist movement was restricted for a period which may cause joint stiffness. Despite those, all patients felt that the time invested was well spent and the function improvement was satisfying.

Two-point discrimination revealed that no significant evidence of nerve harm occurred between pre- and postlengthening. Liverneaux PA concluded that the nerve lengthening stimulates nerve regeneration from five cases of emergency leg replantation with the shortening-lengthening protocols [13]. These findings demonstrated the good biocompatibility of the nerve under slow distraction.

Finally, we recommend regular stump revision of the bone in order to avoid pricking the fingertip during the distraction if the X-ray examination revealed sharp stump of the phalange; preoperative skin enhancement training will make the stump softer for the intending lengthening.

In summary, digital lengthening by distraction and second-stage bone graft is a valuable technique with satisfactory functions and aesthetic results. A distraction rate of 1 mm/day can be performed without nerve harm. Although, due to the bone graft stage, the whole treatment course may be prolonged, it is a worthy and safe procedure without any donor-site morbidity or major complications. Functional improvement gained after distraction still needs further study.

## Figures and Tables

**Table 1:** Basic data of 104 patients included.

All patients	
Number of patients	*104*
Gender	
Male	68
Female	36
Age (years)	
Mean	29.5
Range	6–37
Time delay (months)	
Mean	30
Range	3–144
Number of digits (All patients)	201
Number of digits deficiency (each patient)	
One-digital	44
Two-digital	31
Three-digital	21
Four-digital	8
Finger involved	
Thumb	31
Index finger	59
Middle finger	65
Ring finger	34
Little finger	12
Level of deficiency	
Metacarpophalangeal	6
Proximal phalange	167
Middle phalange	28
Surgery	
Metacarpal lengthening	20
Phalangeal lengthening	181

Time delay = time delay between initial injury and operation in our department.

**Table 2:** Results of distraction lengthening.

	Metacarpal	Phalange
Number	20	181
Elongation		
Average	29.2 mm	18.7 mm
Range	25–40 mm	12–32 mm
Elongation rate (%)		
Average	174.4	184.8
Range	145–202	115–283

**Table 3:** Pre- and postlengthening data.

	Pre	Post	*P* value
STPD	(6.94 ± 0.78) mm	(7.06 ± 0.84) mm	*P* = 0.164 > 0.05
SpO_2_ (%)	96.47 ± 1.23%	96.29 ± 1.34%	*P* = 0.182 > 0.05

STPD = Static two-point discriminations.

*P* value = Pairwise *T*-test value.
